# Suggested Case of Langerhans Cell Histiocytosis in a Cretaceous dinosaur

**DOI:** 10.1038/s41598-020-59192-z

**Published:** 2020-02-10

**Authors:** Bruce M. Rothschild, Darren Tanke, Frank Rühli, Ariel Pokhojaev, Hila May

**Affiliations:** 1 Indiana University, 2401W. University Ave., Muncie, IN 47303 USA; 2 Carnegie Museum, 4400 Forbes Ave., Pittsburgh, PA 44272 USA; 3 Royal Tyrrell Museum of Palaeontology, 1500 N. Dinosaur Trail, Drumheller, AB T0J 0Y0 Canada; 4 Institute of Evolutionary Medicine, University of Zurich, Zurich, Switzerland; 5 Department of Anatomy and Anthropology, Sackler Faculty of Medicine, Tel Aviv University, Tel Aviv, Israel; 6 Dan David Center for Human Evolution and Biohistory Research, Shmunis Family Anthropology Institute, Sackler Faculty of Medicine, Tel Aviv University, Tel Aviv, Israel

**Keywords:** Cancer, Ecology, Zoology, Diseases

## Abstract

Susceptibility to diseases is common to humans and dinosaurs. Since much of the biological history of every living creature is shaped by its diseases, recognizing them in fossilized bone can furnish us with important information on dinosaurs’ physiology and anatomy, as well as on their daily activities and surrounding environment. In the present study, we examined the vertebrae of two humans from skeletal collections with Langerhans Cell Histiocytosis (LCH), a benign osteolytic tumor-like disorder involving mainly the skeleton; they were diagnosed in life, along with two hadrosaur vertebrae with an apparent lesion. Macroscopic and microscopic analyses of the hadrosaur vertebrae were compared to human LCH and to other pathologies observed via an extensive pathological survey of a human skeletal collection, as well as a three-dimensional reconstruction of the lesion and its associated blood vessels from a µCT scan. The hadrosaur pathology findings were indistinguishable from those of humans with LCH, supporting that diagnosis. This report suggests that hadrosaurids had suffered from larger variety of pathologies than previously reported. Furthermore, it seems that LCH may be independent of phylogeny.

## Introduction

Understanding diseases that affected dinosaurs may shed additional light on their biology, daily living, and the environments in which they thrived. Despite the huge time gap between dinosaurs and humans, both were susceptible to diseases that shaped and greatly affected their evolutionary history. Thus, recognition of skeletal manifestations of specific diseases in humans may assist in identifying them in dinosaurs as well. For example, gout was recognized in tyrannosaurids^1^, osteoarthritis in *Iguanodon*^2^, and diffuse idiopathic skeletal hyperostosis (DISH) in *Apatosaurus*^3^. Neoplasms (cancer), albeit more difficult to diagnose (several misclassified cases were published in the past years), have also been identified in dinosaurs^4,5^. Disease occurrence in dinosaurs is very infrequent. When present, however, it can tell us about dinosaurs’ immune systems, metabolic disorders, growth and adaptation to a huge body mass, infections, environment, as well as shed light on their mating patterns and hunting techniques.

Herbivorous hadrosaurs, also referred to as “duck-billed” dinosaurs, have a near-global Late Cretaceous fossil record. They reached a length of over ten meters, weighed several tons, and appear to have lived in large herds. They are particularly well-known in southern Alberta, Canada where individual bones and teeth occur in abundance. In addition, several dozen hadrosaur-dominated bonebeds and hundreds of partial-to-complete skeletons have been discovered. In Dinosaur Provincial Park, examples of hadrosaur osteopathy are so common that more than half a dozen examples can be found daily by an experienced field worker. A number of hadrosaur taxa are known from the Park: the cranially uncrested *Gryposaurus* and *Prosaurolophus* as well as the crested *Corythosaurus* and *Lambeosaurus* are the most common. Trauma, in the form of healing caudal vertebrae (centrum and/or neural spinal crush fractures, related to intraspecific trampling), rib fractures, and osteochondrosis of the pedal phalanges, predominate. Fusions of dorsal and caudal vertebrae are also known to occur. A literature review of the entire Hadrosauridae family appears in Tanke and Rothschild^6^, with reviews and specific case studies of Albertan material appearing in Rothschild and Tanke^7^, Straight *et al*.^8^, Tanke and Rothschild^7^, and Tumarkin *et al*.^9^. Other Albertan hadrosaur-centric studies are currently underway.

Langerhans Cell Histiocytosis (LCH) is a benign osteolytic tumor-like bone lesion that is commonly manifested in the skeletal system in either a unifocal or multifocal form^10,11^; it is the most common of the non-infectious granulomatous bone disorders^12,13^. Histiocytosis is the clonal result of neoplastic mutation and proliferation of hematopoietic stem cell precursors of tissue-resident mononuclear phagocytes (histiocytes) or of precursor dendritic cells^14–17^. Mononuclear phagocytes can be divided into two groups: a monocyte-macrophage group (Erdheim-Chester disease), and a Langerhans (dendritic) cell group^18–20^. The latter group normally functions as antigen processors in the immune system. Since Erdheim-Chester disease produces osteosclerotic lesions^21^, in contrast to the lytic lesions of LCH, it will not be further discussed here.

The most common form of LCH, eosinophilic granuloma, is an isolated bone lesion, most commonly afflicting children and adolescents (between ages 5 and 10), with a slight bias toward males^12,22,23^. Two other syndromes, which are considered as the same disease, yet are less common (up to 20% of cases in children), are the Hand–Schuller–Christian and the Letterer–Siwe diseases. The first includes skull lesions, exophthalmos, and diabetes insipidus; the second includes disseminated lesions involving multiple visceral organs^13,23^.

Although LCH was first described in humans by Thomas Smith in 1865, its etiology and pathophysiology have been debated for many years^12,13^. Some consider it a disorder of the immune system (since myeloid cell line-derived and modulated immunological reactions exist), possibly of viral and other infectious causes (although no pathogen was isolated), or a neoplasia, after identifying clonal proliferation of cells and after its response to chemotherapeutic treatment^24–27^. Advancements in sequencing technology enabled the pathophysiology of LCH to be identified and revealed its association with a genetic mutation (mainly in BRAF or MAP2K1 genes), resulting in MAPK hyperactivation of myeloid precursor cells^28^ (and references therein).

LCH usually appears in the skull, followed by the femur, mandible, pelvis, spine, and ribs. Radiological features of the lesion vary between skeletal parts. For example, in the skull, it appears as a solitary or multiple punched-out lytic lesion without a sclerotic rim, whereas long bones exhibit endosteal scalloping and periosteal reaction. Spinal eosinophilic granuloma accounts for 6.5% to 25% of all skeletal LCH cases^29,30^. It usually appears as a solitary lesion involving the vertebral bodies of the thoracic vertebrae, followed by the lumbar and cervical regions of the spine^22,31–34^. LCH clinical manifestation varies: it can be asymptomatic and discovered as an incidental radiographic finding, or it can be symptomatic with pain, swelling, and tenderness around the lesion. The most common symptoms include neck or back pain, restricted motion of the spine, neurologic symptoms, and deformity^11^. Differential diagnosis includes Ewing sarcoma, osteomyelitis, metastases, round cell tumor, lymphoma, and leukemia^23^.

Recognition of disease in the fossil record is challenging. Although patterns of disease have proven diagnostic (at least when applied to skeletal populations)^35^, examination of isolated elements usually does not allow discrimination, and diagnosis of lytic lesions has been especially problematic^36,37^. Nevertheless, some diseases have reproducible differences in skeletal expression, e.g., metastatic cancer, tuberculosis, fungal disease, and as those documented in the current study, which enable distinguishing between them^4,35,38^. All forms of LCH in clinical cases are diagnosed through bone marrow aspirates or biopsies of lesions. The samples obtained are then tested for the presence of the Langerhans cell phenotype, using immune-histochemical staining or electron microscopy^10,23^. Unfortunately, loss of soft tissues through taphonomic processes in the dinosaurs and through defleshing processes in recent humans precluded applying such immunohistologic methodology at this time, since pertinent soft tissues (e.g., dendritic cells) are usually not preserved^39^.

Non-infectious granulomatous bone disorders have rarely been mentioned as a possibility in the anthropological literature^27,40–43^. The absence of previous reports of non-infectious granulomatous bone disorders in the fossil record may be related to difficulties in distinguishing these disorders from other bone tumors due to meager detailed macroscopic descriptions^44^ as the differences between the various lesions are below the resolution of routine radiological techniques, e.g., plain radiographs and CT^35^. Therefore, systematic surveys of skeletal collections for pathologies whose clinical records are available enables recognizing the apparent uniqueness of lesions and tumors such as granulomatous diseases, and creates a standard for their identification in the archaeological and paleontological records.

A search for pathologies in paleontological skeletal collections revealed unique lesions in a hadrosaur, which differed in character from what we had previously observed in individuals (both human and non-human) with cancer and bone tumors^35,45^. However, these lesions were indistinguishable from those noted in a human with clinically documented LCH.

The aim of the current study was to provide the most reasonable diagnosis for the lesions observed in the hadrosaur vertebrae, considering diagnostic criteria based on a systematic pathological survey of the Terry skeletal collection.

## Materials and Methods

### Human material

Two individuals who were diagnosed in life with LCH (based on histology documentation and clinical manifestations) were included in this study. Both originate from skeletal collections: one is the Terry Collection, which consists mainly of individuals from the St. Louis, Missouri area, who died during the first half of the 20^th^ century; it is housed in the U.S. National Museum of Natural History, the Smithsonian, Washington D.C. The second is the Galler Collection, a pathology reference series dating from the mid-19th through the mid-20th century from the Institute of Evolutionary Medicine, Zurich University; it is housed in the Natural History Museum Basel, Switzerland. A fundamental difference between the Terry and Galler collections should be noted: The former consists of the entire skeleton, whereas the latter generally includes only macroscopically recognized pathological elements. The individual from the Terry collection is a 19-year-old African American male, and the individual from the Galler collection is a 3.5-year-old male (#1328/55).

In addition, a systematic survey of the Terry Collection was carried out to identify which bones are most commonly affected in different pathologies (e.g., LCH, osteosarcoma, Ewing sarcoma, fibrous dysplasia, and multiple enchondroma). The authors received permission to study these human specimens.

### Dinosaur material

**Figure 1:**
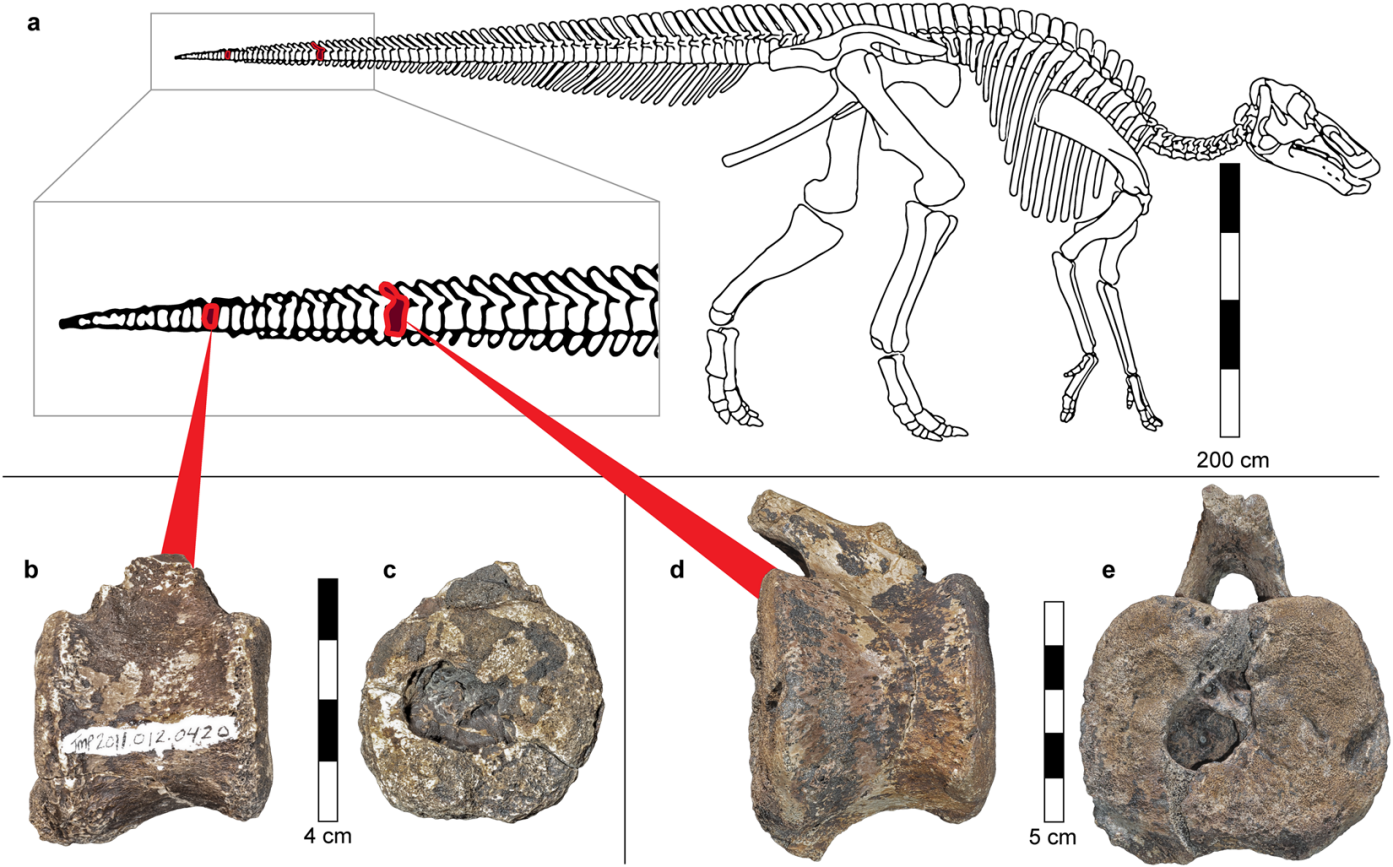
Location of the pathological vertebrae in a hadrosaur skeleton (**a**). Skeleton reconstruction from Campione and Evans^79^. Both small (**b**,**c**) and large pathological vertebrae (**d**,**e**) are from the distal part of the hadrodsaur tail. Note the large oval-shaped cavities that open to the caudal discal surface.

The sample consisted of 11 associated distal caudal hadrosaur centra, not further identifiable as to genus (Royal Tyrrell Museum TMP 2011.012.0420). Of these distal caudal hadrosaur centra, two pathologic vertebrae were identified (Fig. 1). Darren H. Tanke found these vertebrae on the surface in Dinosaur Provincial Park in Alberta, Canada. They are attributed to Quarry 107 (Sternberg, 1950). Of the pathological vertebrae, the smaller one (Fig. 1b,c) is 40.2 mm in height, 40.5 mm in breadth, and 38.0 mm in length. Its total volume is 33,019 mm^3^. The larger of the two (Fig. 1d,e) is 53.2 mm in height (supero-inferiorly), 70.6 mm in breadth (transverse), and 60.0 mm in length (antero-posterior). Its total volume is 134,214 mm^3^.

### Methods used to study human material

Information on the pathologies of the studied samples was obtained from their medical files as well as via macroscopic observation of the pathological bones.

### Methods used to study the dinosaurs’ vertebrae

Preliminary identification of the pathological vertebrae was carried out macroscopically and via epi-illumination microscopy at 20-200x magnification on articular surfaces using a white light-emitting diode (LED) Dino-lite digital microscope (AM7915MZT, Dunwell Tech, Inc., Torrance, CA 90502). To verify the pathology, the vertebrae were scanned via a microfocus X-ray computed tomography system (XT H 225 ST, Nikon Metrology NV, Leuven, Belgium), operated using a 225 kV 225 W reflection target utilizing the following scan parameters: 50 μm, 182 kV, 124 μA, at the Shmunis Family Anthropology Institute, Dan David Center for Human Evolution and Biohistory Research, Sackler Faculty of Medicine, Tel Aviv University. The scan volume was reconstructed using NM CT reconstruction software (CT Pro 3D, v XT 5.3.2, Nikon Metrology, Hertfordshire, UK) and exported in a 16-bit tagged image file format (TIFF). Segmentation and analyses of the vertebrae and the tumors were carried out using Amira (v 6.3, www.fei.com) software. Segmentation was achieved using various semi-automatic tools to reconstruct the 3D mesh surfaces of the tumor, blood vessels, and bone surface. The vertebrae and mass-occupied areas were measured after segmentation.

The dinosaur vertebrae were also photographed at 40x magnification using a stereo microscope (SMZ1270i, Nikon, Tokyo, Japan) equipped with a digital camera (DS-Fi3, Nikon, Tokyo, Japan) at the Department of Anatomy and Anthropology, Dan David Center for Human Evolution and Biohistory Research, Sackler Faculty of Medicine, Tel Aviv University. Image stacks were acquired and processed with the Extended Depth-of-Focus module in NIS-Elements BR software (v 5.2.0, Nikon, Tokyo, Japan, https://www.microscope.healthcare.nikon.com/products/software) to obtain an appropriate depth of field.

## Results

### LCH appearance in vertebrae of humans diagnosed in life with LCH

**Figure 2:**
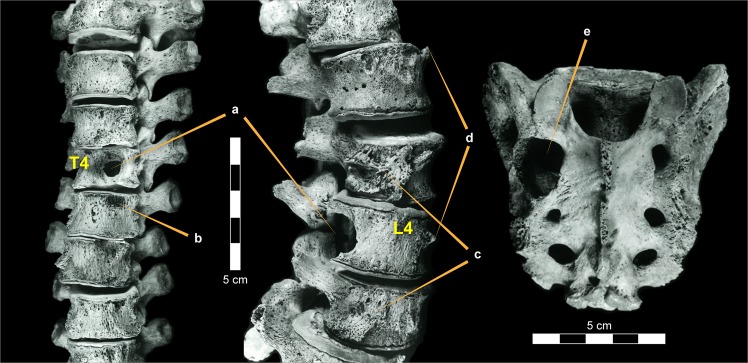
Lesions in human vertebrae in an individual with documented Langerhans Cell Histiocytosis from the Terry collection (individual #129). (**a**) Spheroid-shaped lytic lesion in vertebral bodies, sparing the posterior spinal elements. (**b**) Periosteal reaction on the cortical shells with new bone formation. (**c**) Cortical bone resorption suggesting pressure lesions produced by a paravertebral tumor (eosinophilic granuloma) mass. (**d**) New bone projects from the anterior aspect of bodies. (**e**) A “space occupying mass” in the first left sacral foramina.

The impact of LCH on human vertebra is summarized in Table 1. In both individuals, extensive destructive lesions were noted in the bodies of thoracic vertebrae (Fig. 2). However, the posterior elements of the vertebral body (i.e., elements of the neural arch) were spared. The lytic areas were spherical, with the appearance of an expanding mass, affecting trabeculae (Fig. 2a). They ranged from 10 to 25 mm in diameter. The smooth, round defects with effaced trabeculae had smooth borders and “wrinkled” (crenulated) bases, and were “geographic” in nature (a term applied to a meandering border), where the smaller defects had become contiguous. Scattered areas of resorption were presented on the anterior vertebral surface (Fig. 2b). These apparently represent pressure lesions produced by paravertebral tumor (eosinophilic granuloma) masses. A periosteal reaction was present within the vertebral canal on the posterior aspect of the vertebral body (surrounding the basivertebral foramen), and in the lateral apophyseal region (Fig. 2c). New bone formation appeared on the anterior aspect of the vertebra (Fig. 2d). Although the vertebral endplates were somewhat resistant to destruction (compared with the trabecular bone), expansion of contiguous spheroid masses resulted in endplate perforation.

**Table 1:** Summary of the LCH appearance in the human vertebrae of the individuals from the Terry and Galler collections.

Pathologic appearance	Terry #129	Galler 1328/55
Thoracic vertebra bodies	Destructive lesions	Destructive lesions
Neural arch	Unaffected	Unaffected
Anterior vertebral surface	Areas of resorption	Areas of resorption
Posterior vertebral surface	Periosteal reaction	Periosteal reaction
Thoracic vertebral endplates	Perforated	Perforated
Trabecula in the defected regions	Eroded	Eroded
Shape of the lytic areas	Spherical	Spherical
Size of the lesions	Up to 25 mm	Up to 20 mm
Borders	Meandering	Meandering

The Terry #129 individual exhibited a “space-occupying mass,” expanded to a diameter of 25 mm, at the location of the left upper posterior sacral foramen (Fig. 2e). Visible internal surface trabeculae were affected, producing a smooth surface. Radiological examination of Galler 1328/55 vertebra revealed lytic lesions (limited to vertebral centra), usually with sharp “zones of transition.” Minimal sclerosis was also noted in one of the affected vertebrae, and minimal new bone was present at the anterior aspect of several centra.

Table 2 shows the prevalence of neoplasia shown by various skeletal elements in different pathologies, based on the Terry Collection, to aid in the differential diagnosis of tumors in skeletal material.

**Table 2:** Pathological survey^a^ of the Terry collection. Prevalence (%) of individuals affected by multicentric bone neoplasia, by bone.

Bone affected	LCH (T-129^b^)	Eosinophilic Granuloma	Osteosarcoma	Ewing Sarcoma	Metastatic Carcinoma	Leukemia
Innominate	+	10–12	7–8	12–20	41	2–85
Femur	+	13–32	44–46	22–27	25	22–75
Tibia	+	4–18	17–21	9–11	3	6–50
Fibula	−	1–5	3–5	8–9	0	2
Foot	+	1–2	1	3	0	2–8
Scapula	+	5–6	1	5	6	2
Humerus	+	5–11	15	8–10	10	0–55
Radius	+	1–3	0–1	1–2	0	0–65
Ulna	−	0	0–1	1–2	0	2–65
Hand	−	0	0–1	1	0	1–25
Skull	+	5–27	1–5	1	5	6
Mandible	−	3–11	2–4	1	0	3–58
Vertebrae	+	18–25	1	1–6	69	4
Sternum	−	1	1	0–1	0	1
Clavicle	−	2–4	1	1–2	0	2
Ribs	+	1–8	1–2	8–11	25	6

^a^Lesion identification derived from Dabska and Buraczewski^65^, Dahlin and Ivins^66^, Shapiro^70^, Spjut *et al*.^72^, Resnick and Niwayama^38^, Kransdorf *et al*.^68^, Huvos^71^, Kilpatrick *et al*.^79^, and Rotschild and Rotschild^60^.

^b^In the Terry collection, LCH was present only in T-129. Thus, ‘+’ indicates bone affected and ‘−’ unaffected. The Galler specimen is not included in this table since his complete skeleton was not available for assessment.

### Osseous changes in hadrosaur TMP 2011.012.0420

**Figure 3:**
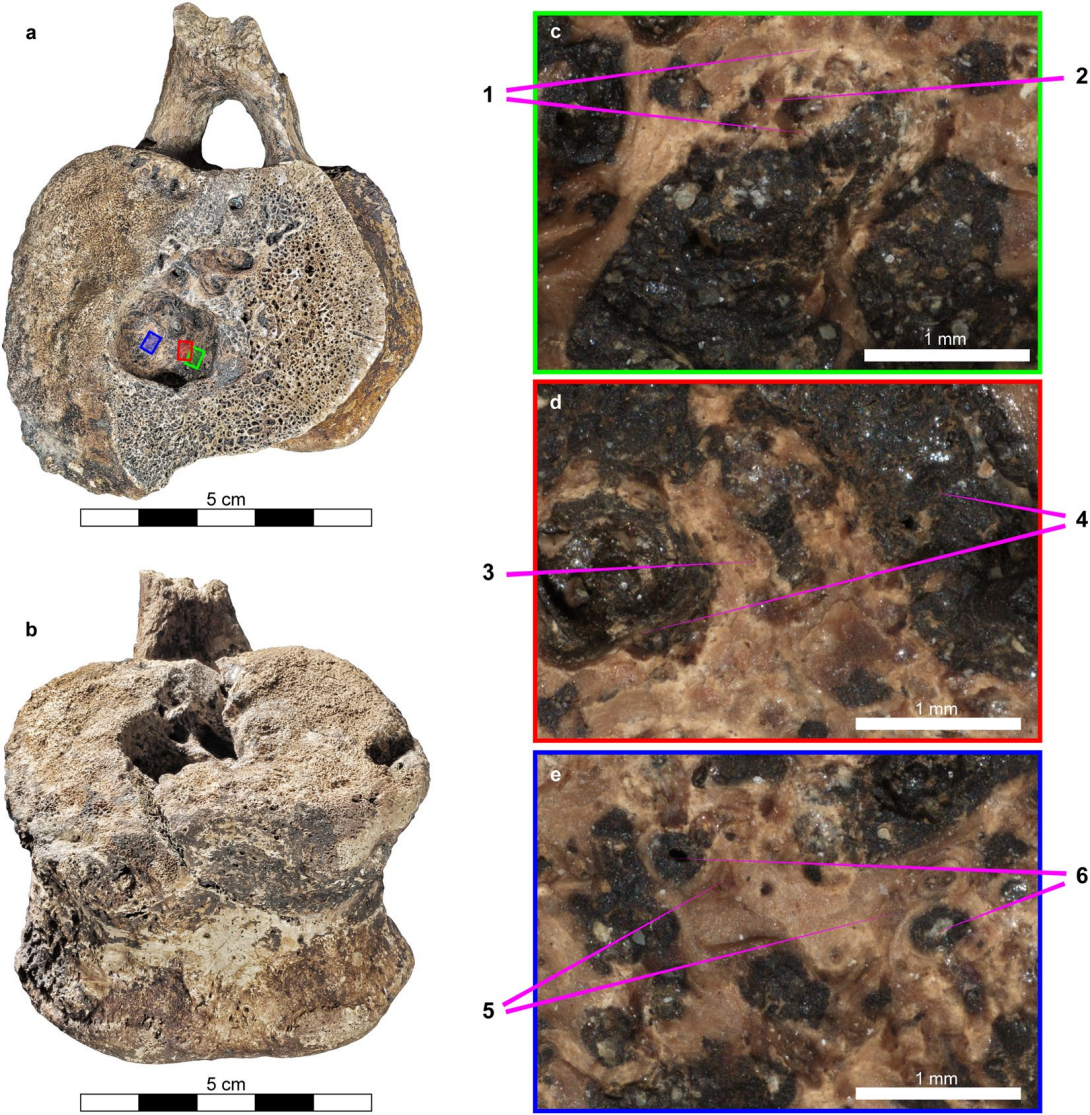
Photograph of the large hadrosaur vertebra in caudal view (the vertebrae was cut lateral to the lesion to examine its inner structure) (**a**) and a ventro-caudal view (**b**) (photographs taken via Canon EOS 5D MARK III), Note that the opening is oval, and its rim is smooth. Microscopic examination of the bottom of the lesion (magnification X40, Nikon SMZ1270i, digital camera Nikon DS-Fi3, Tokyo, Japan) (**c**–**e**) show a clear “zone of transition” (1) surrounding “a zone of resorption” (2) adjacent to the “zone of resorption” and effaced trabeculae (3), big vessels were identified (4). Scattered areas of resorption (5) and numerous small blood vessels (6) are also visible.

**Figure 4:**
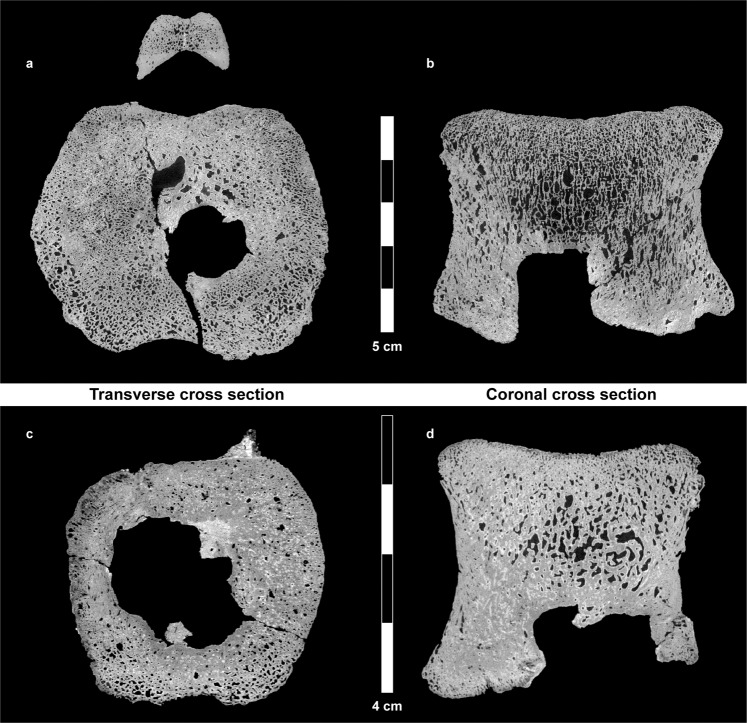
Transverse (horizontal) and coronal cross sections retrieved from the µCT scans of the large (**a**,**b**) and small (**c**,**d**) vertebrae. In some areas, there is clear evidence of bone remodeling adjacent to the tumor (sclerotic margins). Multiple lesions coalesce, thus creating a single lesion with well-circumscribed margins, having the “geographic” pattern (a term applied to a meandering border).

Macroscopic examination of hadrosaur vertebrae (TMP 2011.012.0420) revealed geographic vertebral defects, smooth-walled, with multiple smooth extensions. Serpentine, smooth-bordered erosions with “wrinkled” bases, gave the impression of a “space-occupying mass.” Subjacent trabeculae were effaced, with no evidence of new bone formation or “blunting” (Fig. 3a,b). However, posterior vertebral elements (spinous and transverse processes) were spared (Fig. 3a). The spheroidal cavities in both vertebrae are large and open toward the caudal aspect of the vertebra (Fig. 3a,b). The opening is oval in shape and the rim is smooth, with no evidence of bone remodeling (Fig. 3a,b). Changes in trabeculae within the cavity are considerable (sclerotic and thicker) (Fig. 4). The inner walls are smooth, with cavities of various sizes expanding laterally (Fig. 4). Numerous openings, also of various sizes, are visible mainly at the bottom of the cavity.

Microscopic examination revealed a clear “zone of transition” (Fig. 3c) between the normal and the damaged bones. This shows a well-defined margin for the damaged area (“the zone of resorption”) (Fig. 3c). Additionally, large blood vessels were identified adjacent to effaced trabeculae (Fig. 3d), as well as numerous small blood vessels (Fig. 3e) and small scattered areas of resorption (Fig. 3e).

**Figure 5:**
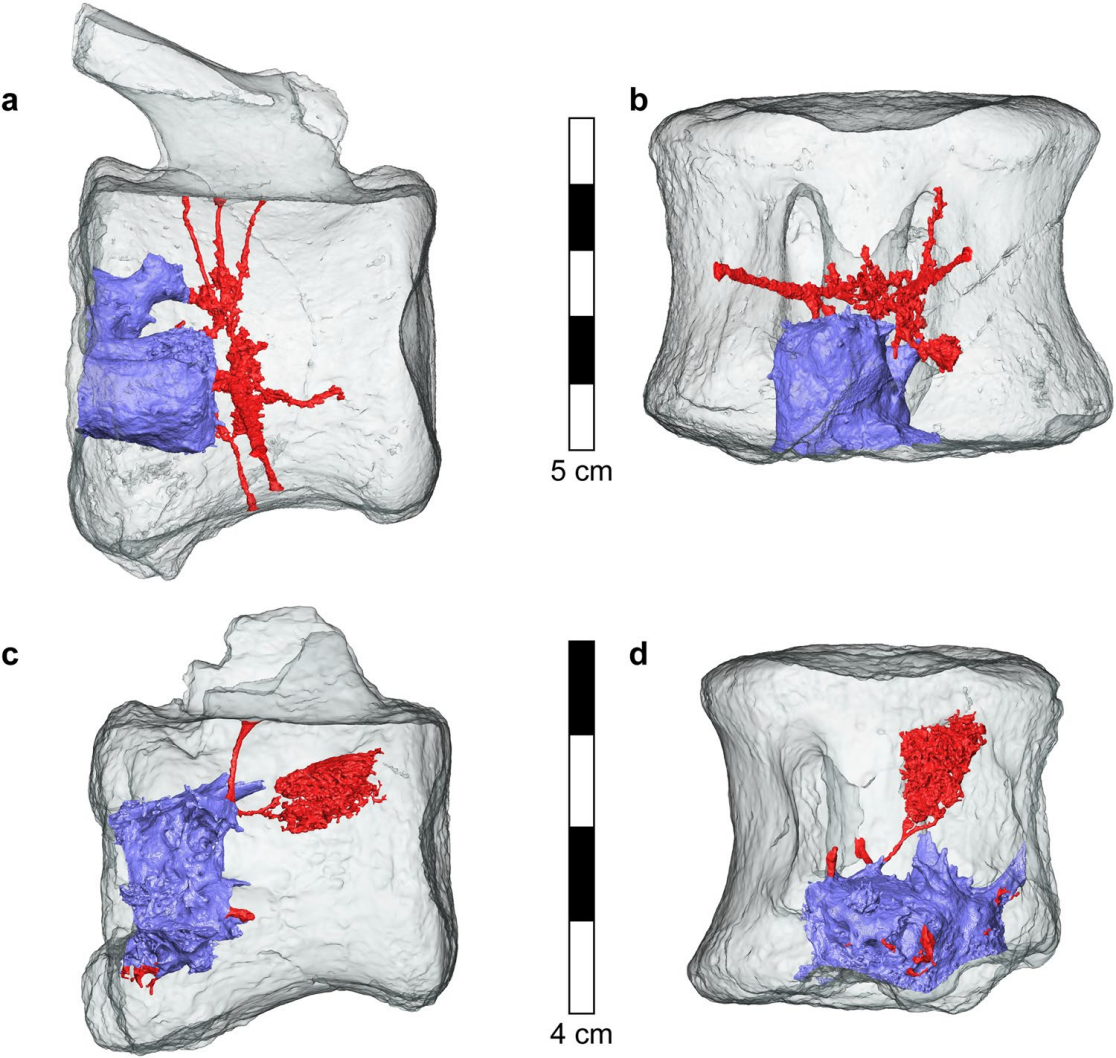
Three-dimensional reconstruction of the mesh surface of the tumors and associated blood vessels in the large (**a**,**b**) and small (**c**,**d**) hadrosaur vertebrae. The tumors (purple) in both hadrosaur vertebrae are located at the lower (caudal) central part of the vertebral bodies. Numerous blood vessels (red) of various size reach the tumors. Tumor coalescence is evident. The tumor surface is irregular, with many small and large branches.

The 3D reconstruction of the cavity revealed the presence of a space-occupying mass at the center of the vertebral bodies. The mass, generally spheroidal in shape, nevertheless possesses numerous extensions of varying size. Many blood vessels reach the mass, some directly via the basivertebral opening (Fig. 5). Lesions in both vertebrae were large, occupying a considerable part of the vertebral body volume (Fig. 5): the larger mass was 20.6 mm in height, 17.0 mm in breadth, and 17.7 mm in length. The total volume of the tumor was 5411 mm^3^, occupying 4% of the total volume of the vertebra. The smaller mass was 11.4 mm in height, 21.0 mm in breadth, and 17.7 in length, The total volume of this tumor was 2440 mm^3^, occupying 7.4% of the total volume of the vertebra.

## Discussion

This is neither the first report of LCH in a veterinary record^46,47^ nor the first report of tumor/tumor-like pathology in dinosaurs^48,49^. Hadrosaurs were unique among dinosaurs regarding their susceptibility to neoplastic processes, both benign and malignant^6,48^. Nevertheless, this study is the first to suggest a case of LCH in the fossil record, and the first to provide evidence-based criteria (predicated on findings in individuals diagnosed in life) for its recognition in skeletons, when soft tissue structures cannot be assessed. We feel confident in comparing the appearance of LCH in humans and dinosaurs, since monocytes and lymphocytes (in contrast to erythrocytes and neutrophils) of reptiles and birds have morphology and function similar to those of mammals^50–52^.

LCH produces well-defined, eccentrically shaped lytic bone defects^13,53^. Spinal involvement by LCH mainly affects thoracic vertebral bodies (centra), sometimes resulting in anterior wedging or a near collapse, with a “vertebra plana” appearance. Posterior elements are rarely involved^53,54^. This pattern of vertebral involvement is noted in Terry #129 and Galler 1328/55, as well as TMP 2011.012.0420^7^. The coalescence of multiple lesions (thus creating the “geographic” appearance)^13,55–57^, as well as the “space-occupying masses” and effaced trabeculae (with or without periosteal reaction) appear to reproducibly define LCH; they were present in Terry #129 and Galler 1328/55, as well as TMP 2011.012.0420. These characteristics are also notable in MRI images of a vertebra of a 47-year-old male who was diagnosed with LCH^58^.

An apparently unique feature is effaced trabeculae, occasionally with wrinkled bases. The lesions were different in appearance from those seen in metastatic cancer, tuberculosis, and fungal disease^35,59,60^, and have not been observed in any other lytic process.

Differential diagnosis (other diagnostic considerations) of LCH includes tuberculosis, fungal disease, brucellosis, echinococcosis (dog tapeworm), metastatic cancer, multiple myeloma, lymphoma, leukemia, hyperparathyroidism, osteomyelitis, and bone tumors (Table 2)^23,61^. Schmorl’s nodes are not included in the differential diagnosis for two main reasons: (1) They expand into vertebral endplates from the disk space rather than expanding from the vertebral body center outward through the surface, as observed in our case. (2) Hadrosaurs do not have intervertebral disks; their vertebral centra articulations are synovial lined joints^62^.

Bone destruction with well-defined margins is generally a sign of a relatively benign process. The important characteristic is the so-called “zone of transition” between normal and abnormal bone. Benign processes tend to have a very sharp or thin “zone of transition.” Less well-demarcated “zones of transition” are more characteristic of malignant bone tumors and infectious diseases.

While LCH may invade a joint by contiguous growth, tuberculosis is predominantly a disease of joints^61,63^. The most characteristic aspect of lytic lesions in tuberculosis is the presence of smooth “zones of resorption”. This is in contrast to the “space-occupying mass” appearance noted in LCH. The appearance of LCH also differs from the “fronts of resorption” perforated by blunt 1 × 2-mm spicules of new bone, apparently characteristic of fungal disease^56^. Brucellosis produces a very characteristic anterior vertebral articulating surface groove^35^. Echinococcal hydatid cysts produce expansile lytic lesions with small cystic outpocketing^61^. Subchondral (so-called degenerative) cysts are neither multilocular nor multilevel phenomena, and do not have a “geographic” appearance^61^.

Among bone tumors affecting the vertebra, LCH can be distinguished from: the expansile, soap bubble appearance of aneurysmal bone cysts that have thin, eggshell-like bony margins that predominantly affect posterior elements of vertebrae; the radiolucent nidus of osteoid osteoma; the large expansile lytic metaphyseal lesions of osteoblastomas; the solitary, vertebral nature of giant cell tumors (with cortical thinning and delicate trabeculae) and chondroblastoma that demonstrate epiphyseal popcorn-shaped calcification characteristics of the latter; the non-ossifying and desmoplastic fibromas (which are superficial and ovoid); the ground glass appearance of fibrous dysplasia (which tends to be much more widespread in the polyostotic form and is associated with scalloping of endosteal bone); the ill-defined margins of Ewing sarcoma and osteosarcoma, which usually do not affect vertebrae; the vascular tumors (e.g., hemangiomas), which may be bubbly or have characteristic linear residual trabeculae; and the lipid cell tumors, which are lobulated and have internal bridging^35,61,64–73^.

Hyperparathyroidism rarely produces skeletal alterations^35,61^. While most are highly characteristic, “brown tumors” of that disease may cause confusion. Recognized radiologically, they are internal osseous processes, which would not be anticipated as a source of confusion with the surface lytic processes of LCH. Furthermore, osteoclast-based resorption (characteristic of brown tumors) produces “fronts of resorption”^74^, not “space occupying masses” (characteristic of LCH).

The absence of remodeled trabeculae and of the buttressing of subjacent trabeculae appear to distinguish the lesions of LCH from those of metastatic cancer (characterized by trabecular remodeling and buttressing). Although discrete lytic lesions in individuals with metastatic cancer have the “space occupying” appearance^59,61^, the presence of a wrinkled base appears to be specific for LCH.

Poorly demarcated “zones of transition,” with borders merging imperceptibly with the surrounding healthy bone, are found in Ewing’s sarcoma, often associated with a laminated or with a “hair-on-end” periosteal reaction^61,71,72^. Although “vertebra plana” can occur with Ewing’s sarcoma, the lesion is more aggressive and ragged in appearance, in contrast to the “space occupying mass” appearance of LCH.

Solid tumors of white blood cells (e.g., lymphomas or leukemia) produce a permeative or “moth-eaten” appearance, eventually disrupting the cortex^1^. An “onion-skin” type of periosteal lamination may be present^75^, contrary to what is noted in LCH. Multiple myeloma may produce a “space occupying mass” appearance, but it is uniquely expansile, penetrating cortical as well as trabecular bone. This results in a fully spherical appearance^76^, contrary to observations in LCH.

Whereas malignant histiocytosis is reported as an aggressive disease in the veterinary literature for dogs and cats^77,78^, it is a very different disorder from the benign process of LCH that most probably affected the hadrosaur in the current paper. There are, however, reports of LCH in tigers *Panthera tigris*)^47^ and a tree shrew (*Tupaia belangeri*)^46^.

## Conclusions

Based on our radiographic analysis of the hadroseaur vertebrae and the extensive comparison with various human pathologies, we suggest that this case is the first LCH disease recognized in a dinosaur. This report extends the spectrum of pathological processes with which these denizens of the Cretaceous Period were afflicted, and suggests that pathological processes are independent of phylogeny and time.
